# Who Enjoys Teaching, and When? Between- and Within-Person Evidence on Teachers’ Appraisal-Emotion Links

**DOI:** 10.3389/fpsyg.2020.01092

**Published:** 2020-06-19

**Authors:** Anne C. Frenzel, Daniel Fiedler, Anton K. G. Marx, Corinna Reck, Reinhard Pekrun

**Affiliations:** ^1^Department of Psychology, University of Munich, Munich, Germany; ^2^Department of Psychology, University of Essex, Colchester, United Kingdom; ^3^Institute for Positive Psychology and Education, Australian Catholic University, Sydney, NSW, Australia

**Keywords:** teacher emotions, teacher goals, appraisals, multilevel regression, between-person analyses, within-person analyses

## Abstract

Testing assumptions proposed by Frenzel’s reciprocal model of teacher emotions (e.g., Frenzel, 2014), this study explored relations between teachers’ appraisals concerning the attainment and importance of their teaching goals, and their emotions. Specifically, we addressed teachers’ goals of high student performance, motivation, discipline, and high-quality teacher–student relationship and three key discrete emotions, namely, enjoyment, anger, and anxiety, during teaching. We had 244 secondary school teachers (70.1% female) self-report their goal attainment and importance appraisals and emotional experiences with respect to up to three different classes they currently taught. Results from single- and two-level multivariate multiple regression analyses largely supported the relevance of the goal attainment appraisals for teachers’ emotions both on the between-person and the within-person level. Goal importance appraisals proved to be of secondary relevance. On the between-person level, those teachers who positively appraised the attainment of motivation, discipline, and teacher–student relationship quality proved to report more enjoyment and less anxiety and anger. On the within-person level, teachers reported enjoying teaching those classes more, which they perceived as better performing, more motivated and disciplined, and with whom they had a better relationship. Anger and anxiety were negatively linked to appraisals pertaining to the attainment of discipline and teacher–student relationship quality. Across both analysis perspectives, teacher–student relationship quality attainment showed particularly strong links with all three emotions. Because teachers’ subjective evaluations regarding student behaviors were shown to be highly relevant for their emotions, we conclude that teachers could be supported in modifying their emotional experiences through cognitive reappraisal. Interventions targeting teachers’ relationships with students, and their cognitive judgments thereof, seem particularly promising.

## Introduction

In the present contribution, we conceptualize emotions as multicomponential constructs, jointly activated by how events are interpreted (e.g., Scherer, 2000). We further take on a discrete emotions perspective, differentiating between conceptually separable “packets of experience,” which are characterized by different parameters of the emotion-defining components, as well as different appraisal constellations (e.g., Barrett et al., 2009). Teacher emotions, in particular, are conceptualized as emotions experienced in the context of their professional engagement as teachers. Teacher emotions have been shown to be highly relevant not only for important student outcomes but also for teachers themselves. By and large, pleasant teacher emotions seem to be integral parts of, and conducive to, a range of desirable outcomes, including teaching enthusiasm, supportive teaching strategies, and well-being among teachers (Kunter et al., 2011; Frenzel et al., 2016; Keller et al., 2016; Chen, 2019; Russo et al., 2020), as well as student motivation, enjoyment of learning, self-regulated learning, and performance (Babad, 2007; Beilock et al., 2010; Frenzel et al., 2016; Keller et al., 2016; Banerjee et al., 2017). Unpleasant emotions tend to be linked to undesirable outcomes, including (intentions to) dropout, burnout, and problematic teaching strategies among teachers (e.g., Skaalvik and Skaalvik, 2011; Rothland, 2013; Seiz et al., 2015; Frenzel et al., 2016; Chen, 2019), and disruptive behavior, anxiety, and decreased achievement among students (Chang, 2013; Arens and Morin, 2016; Klusmann et al., 2016; Aldrup et al., 2018). Teacher anger may be one notable exception: If expressed adequately after student failure, anger can have positive effects on students as it signals high expectations for the students (Butler, 1994; Frenzel and Taxer, 2018). Overall, it seems desirable that teachers be supported so that they experience more pleasant and less unpleasant emotions. In order to derive scientifically sound ideas for how this can be achieved, insight into the antecedents and correlates of teachers’ emotional experiences is essential. This is the key goal of the present contribution.

## Theoretical Background

**FIGURE 1:**
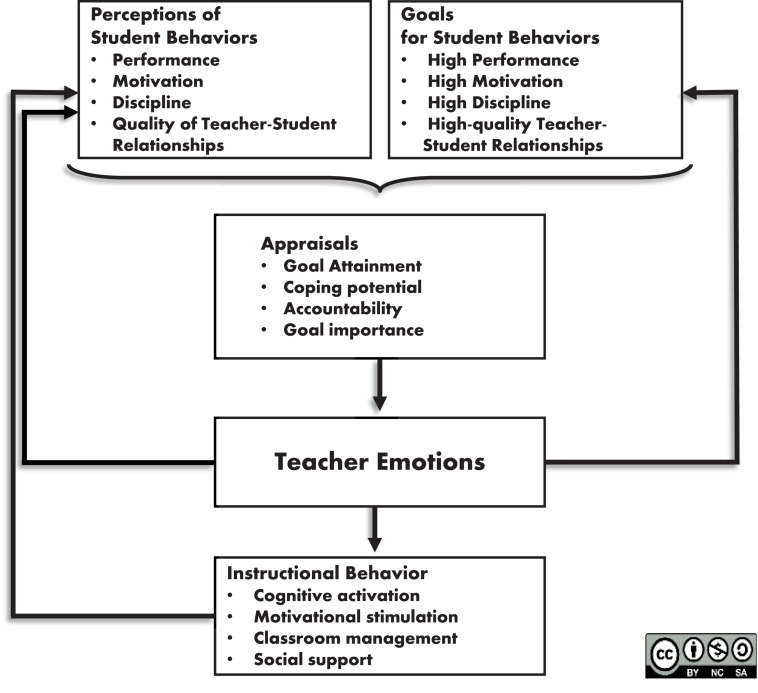
Frenzel’s reciprocal model of teacher emotions (adapted from Frenzel, 2014). This Figure is licensed under CC BY-NC-SA 4.0.

Our theoretical reasoning about the arousal of emotions is grounded in appraisal theory (Roseman and Smith, 2001; Scherer et al., 2001; Ellsworth, 2013; Moors et al., 2013). Appraisal theory claims that it is not events *per se* that arouse emotions, but the individuals’ cognitive interpretations of those events. For example, a student would not fear a test *per se*, but test anxiety will be aroused once the student judges the chances of failing the test as sufficiently high, and their potential to avoid this failure as low. In the context of teaching, appraisal theory has been used in Frenzel’s reciprocal model of teacher emotions (Frenzel et al., 2009; Frenzel, 2014; Jacob et al., 2017). This model (Figure 1) describes appraisal antecedents of teachers’ emotions, as well as the effects of teacher emotions for student behaviors, and proclaims that the latter are linked through reciprocal causation through recursive feedback loops. In short, this model proposes that teachers have certain key goals they strive to attain during their teaching, and they continually make judgments pertaining to those goals based on their perceptions of their students’ behaviors, hence appraising the current classroom situation, resulting in differential emotional experiences during teaching. Those emotions should have effects on teachers’ classroom behaviors, which in turn should be recursively linked with students’ behaviors and thus teachers’ appraisals thereof. The section of this model which addresses the appraisal antecedents of teachers’ emotions, serves as theoretical framework for the present study.

Appraisal theory has been deemed meaningful as a lens through which to understand teacher emotions by a range of authors (e.g., Chang, 2009; Tsouloupas et al., 2010; Fried et al., 2015). In line with Butler (2007), who argued that “the school is an achievement arena not only for students but also for teachers who presumably strive to succeed at their job” (p. 242), Frenzel et al. (2009) proposed that teachers’ appraisals about the success or failure regarding their teaching job are key to understanding teacher emotions. More specifically, according to this model, teacher emotions are elicited based on teachers’ appraisals concerning their classroom goals.

Therefore, it is essential to understand which goals teachers may strive to achieve during their teaching. The identification of potential key teaching goals as proposed within Frenzel’s reciprocal model of teacher emotions (Frenzel et al., 2009; Frenzel, 2014; Jacob et al., 2017) was informed by different theoretical approaches. First, by Tschannen-Moran and Woolfolk Hoy’s (2001) threefold conceptualization of teaching efficacy comprising efficacy for instruction, student involvement, and classroom management. In addition, it considered existing models of teaching effectiveness (Rakoczy et al., 2007; Klieme et al., 2009), which propose that key quality dimensions of instruction are cognitive activation, classroom management, clarity and structure, and supportive climate. Third, it took into account Butler’s notion of teacher relational goals (i.e., their striving to connect with children; Butler, 2012; Butler and Shibaz, 2014). Integrating across those approaches, Frenzel and colleagues (Frenzel, 2014; Jacob et al., 2017) suggested that most teachers should have four key goals they strive to attain during their teaching, which are (1) high student performance, (2) high student motivation; (3) high student discipline^1^, and (4) high quality of teacher–student relationships. With those goals in mind, teachers are proposed to continually observe their students’ behaviors, and make corresponding appraisals. Based on these propositions, we hypothesized in the present study that teacher appraisals pertaining to the attainment and importance of high levels of student performance, motivation, discipline, and high-quality teacher–student relationships would be particularly relevant for teachers’ emotional experiences.

Furthermore, teachers’ judgments pertaining to those goals based on their perceptions of their students’ behaviors should form the basis of their appraisals (denoted by the curly bracket in Figure 1). Four different appraisals are proposed to be relevant for teachers (Frenzel et al., 2009; Frenzel, 2014; Jacob et al., 2017): (1) goal attainment (or goal consistency/conduciveness) in terms of judgments to what degree the goal is met, (2) coping potential in terms of judgments of feeling capable of solving the problem in case of goal non-attainment, (3) accountability in terms of judgments who is responsible for the attainment or non-attainment of a goal, and (4) goal importance in terms of the relevance attached to the attainment of the particular goal.

The present contribution focuses on goal attainment and goal importance appraisals. We propose that goal attainment appraisals should be positively linked with the experience of positive emotions and negatively linked with negative emotions. That is, teachers should experience more positive emotions if they experience higher success in attaining their goals, and they should experience more negative emotions, the more they sense they are failing to attain their goals. Goal importance appraisals should further boost the intensity of teachers’ emotions, in terms of an interaction with goal attainment appraisals: The more a teacher deems it important to achieve a particular goal, the stronger their positive emotions should be in case of goal attainment, and the stronger their negative emotions should be in the case of non-attainment of a goal. Such reasoning about multiplicative combinations of goal attainment and goal importance appraisals has a long-standing theoretical history in the context of achievement motivation (Feather, 1982; Nagengast et al., 2016) and achievement emotions (Pekrun, 2006, 2018).

## Prior Empirical Evidence

Qualitative research has long been emphasizing the role of teacher perceptions of goal attainment for their emotions. Specifically, attaining high levels of performance, motivation, discipline, and high-quality teacher–student relationships has consistently been mentioned in this body of qualitative literature (Goldstein and Lake, 2000; Hargreaves, 2000; Zembylas, 2002; Winograd, 2003; Sutton, 2007; Hagenauer and Volet, 2014; Khajavy et al., 2018). There also are a handful of studies that quantitatively investigated links between teachers’ goal attainment appraisals and their emotions.

Frenzel et al. (2009) explored the links between teacher judgments of student performance, motivation, and discipline, on the one hand, and their experiences of enjoyment, anger, and anxiety, on the other. Teacher reports were recorded through questionnaires referring to one of the teachers’ classes and through diaries where teachers made their judgments retrospectively directly at the end of multiple lessons across 2 weeks of teaching. Multiple regression results showed that the attainment of student motivation and discipline was significantly linked with teacher reports of enjoyment, anger, and anxiety, whereas the attainment of high student performance did not have any additional predictive power. The lesson diary analyses suggested that the attainment of all three goals accounted for significant amounts of the variance of the teachers’ daily emotional experiences, with discipline attainment being particularly relevant for anger and motivation attainment being particularly relevant for enjoyment. The authors concluded that teachers’ emotions fluctuate strongly from lesson to lesson and that teacher appraisals are highly relevant in explaining these fluctuations.

Hagenauer et al. (2015) explored links between teachers’ perceived success in promoting high student motivation (referred to by these authors as engagement), discipline, and high-quality teacher–student relationships (referred by these authors as interpersonal teacher–student relationships), on the one hand, and teachers’ enjoyment, anger, and anxiety, on the other. They used self-report questionnaires focusing on one class the teachers were currently teaching from a sample of 132 secondary school teachers. Latent multiple regression analyses showed that attainment of all three goals proved to have significant links with each of the three emotions, with one exception—student engagement being unrelated to teacher reports of anxiety. High-quality teacher–student relationships were most strongly linked with teacher enjoyment (positive relation) and anxiety (negative relation), whereas discipline showed the strongest links with anger (negative relation). They concluded that teacher–student relationships play a particularly important role for teachers’ emotional experiences in class.

Becker et al. (2015) applied a lesson diary approach in a sample of 39 secondary mathematics teachers and their 758 students to assess both student and teacher perspectives on classroom events. They assessed student reports of their motivation and discipline and teacher reports of their enjoyment and anger. Additionally, they obtained teachers’ general goal conduciveness appraisals (operationalized as “In this lesson, students’ behavior was beneficial for my lesson goals”) and coping potential appraisals (operationalized as “In this lesson, I felt like I had everything under control”). They found that the higher students’ motivation and discipline, the more the teachers appraised the situation as being conducive to their lesson goals and that they were in control, which in turn jointly positively predicted their enjoyment, and negatively predicted their anger, during the lessons. These findings further supported claims that appraisals pertaining to student behaviors are linked with teachers’ emotional experiences.

There is also scattered empirical evidence on the relevance of the multiplicative combination of control and value appraisals (which are conceptually similar with what is denoted here as goal attainment and goal importance appraisals), above and beyond first-order effects of the control and value appraisals, for students’ emotions (Goetz et al., 2010; Bieg et al., 2013). For example, Bieg et al. (2013) showed that the combined effect of low control and high value resulted in more intense feelings of anxiety among students. Goetz et al. (2010) could show that students’ enjoyment, pride, and contentment were particularly elevated when both control and value of a situation were appraised as high, as predicted by Pekrun’s (2006, 2018) control-value theory. In other words, those studies showed that control appraisals were particularly relevant for students’ emotional experiences if the situation was appraised as important. So far, no study seems to have explored the predictive power of the multiplicative link between goal attainment appraisals and goal importance appraisals for teachers’ emotions.

## The Present Study

The present study focuses on the three discrete emotions enjoyment, anger, and anxiety, as well as the appraisals of goal attainment and goal importance. It operationalizes emotions as conceptualized in Frenzel et al.’s (2016) Teacher Emotions Scales, taking a trait-based, class-specific approach to measuring emotions by asking teachers how they “generally feel” when teaching a particular group of students. Furthermore, we obtained teachers’ judgments regarding the attainment of desirable levels of student performance, motivation, discipline, and high-quality teacher–student relationships, as well as teachers’ judgments of how important it was for them to achieve those goals.

An important feature of the present study is that it was purposefully designed to explore the proposed links between appraisals and emotions both from a between-person and from a within-person perspective (Murayama et al., 2017). The between-person perspective implies exploring the covariation between the reported levels of emotional experiences and the goal attainment judgments across teachers. The within-person perspective implies exploring the covariation between the multiple emotion ratings and multiple goal attainment appraisals within each teacher. From a between-person perspective, we asked, for example, if one teacher experiences to be more successful in attaining student discipline than another teacher, will this teacher also enjoy teaching more than the other teacher? In other words, this analysis approach allows exploring *who*—across a population of teachers—experiences most enjoyment, anger, and anxiety during teaching.

In order to additionally realize a within-person analysis perspective in our study, we had teachers to report not only about one single group of students, but additionally about up to two more classes they were currently teaching. Thus, we could ask, for example, if one teacher experiences to be more successful in attaining student discipline in one of his classes more than in another one of his classes, will this teacher also enjoy teaching this class more than the other class? In other words, this analysis approach allows exploring *when* a single teacher experiences most enjoyment, anger, and anxiety during teaching.

It is worth noting that the proposed psychological theory underlying the present research (appraisal theory) focuses on intraindividual psychological functioning (Goetz et al., 2016)—both emotions and appraisals are supposed to be highly individualized, context-specific phenomena, which can strongly vary from situation to situation, and which are shaped by contextual factors. As such, the covariation between appraisals and emotions is theoretically proclaimed to be located at the within-person level.

Intriguingly, despite a clear within-person focus of many psychological theories, a large majority of the existing research has been conducted using a between-person approach. However, there is a multitude of factors that can render between-person and within-person relations non-equivalent, and results will converge only if specific assumptions are met (see Hamaker et al., 2005; Voelkle et al., 2014; Murayama et al., 2017; Fisher et al., 2018). Given that prior research exploring emotions from both a between- and a within-person perspective tended to have shown equivalent results (e.g., Goetz et al., 2016; Murayama et al., 2017), we also expected convergence of findings across both approaches in the present study.

Concerning the question of the existence and size of within-teacher variance in emotional experience, Frenzel et al. (2015) have shown that teachers’ enjoyment, anger, and anxiety indeed vary considerably within teachers, and that some of this variability is due the various groups of students they teach. This is in line with Raudenbush et al. (1992) and Ross et al. (1996) findings on the group specificity of teaching self-efficacy and with interview data reported by Hargreaves (2000), indicating that teachers’ emotional experiences are related to factors characterizing the specific group of students taught. However, we know of no study that explored the intraindividual variability of goal attainment appraisals among teachers and their within-person covariation with teachers’ emotions.

In summary, the present study addressed the following research questions:

1. How much variance of teachers’ enjoyment, anger, and anxiety is explained by teachers’ goal attainment appraisals
  (a) from a within-teacher perspective?
  (b) from a between-person perspective?
2. Does the additional consideration of goal importance appraisals result in explaining further significant proportions of variance?

With respect to Research Question 1, we expected that goal attainment appraisals should be positively related with enjoyment and negatively related with anxiety and anger. We expected significant links for each of the four goals (high student performance, motivation, and discipline and high-quality teacher–student relationships). Furthermore, based on scattered prior findings, we anticipated the attainment of high-quality teacher–student relationships to be particularly relevant for all of the emotions and the attainment of high discipline to be additionally particularly relevant for anger. However, it is worth noting that prior research so far has not yet considered the attainment of all four goals as joint predictors of emotions. Therefore, the present study provides novel insight into the relative emotional relevance of each of the four postulated teaching goals.

With respect to Research Question 2, we expected that teachers’ enjoyment, anger, and anxiety are more strongly affected by their goal attainment appraisals if those goals are important to them. We did not expect that the goal importance appraisals *per se* would be linked with the emotional experiences (e.g., a teacher’s enjoyment should not generally be higher or lower depending on how important it is for a teacher that the students perform well, regardless of attainment of this goal). However, we did expect that goal attainment appraisals and goal importance appraisals would interact in their effects on the emotions. For example, a teacher’s enjoyment should be boosted if they deem student performance as important, coupled with their judgment that their class is doing particularly well. Those assumptions are in line with expectancy-value and control-value theoretical claims as brought forward for achievement motivation and emotion and empirical findings on students’ emotions in this context. However, no study so far seems to have explored the impact of goal importance appraisals for teachers’ emotional experiences.

## Materials and Methods

### Sample and Procedure

Participants of this study were 244 secondary school teachers (70.1% female) from different southern German states (predominantly Bavaria, 81.1%; Baden-Wurttemberg, 11.9%; and other, 7.0%) who taught at more than 40 different secondary schools. Specifically, 15.6% taught at lower-track schools of the German secondary school system (Hauptschule), 18.9% at medium-track schools (Realschule), and at 55.3% in high-track schools (Gymnasium); 10.2% taught at different tracks simultaneously or at other types of secondary schools such as vocational schools^2^. Teachers were on average 42.9 years old (SD = 10.9, min = 27, and max = 65 years), had on average 13.25 years of teaching experience (SD = 10.4, min = 0.5, and max = 40 years), and taught a wide range of subjects. In total, 483 teachers had been invited to participate in our study. The questionnaire return rate was thus 50.5%, which is highly satisfactory as it exceeds that of earlier studies (Metler, 2003, 21%; Taxer and Frenzel, 2015, 32.8%). Teacher recruitment occurred on a school level through convenience sampling by trained student study administrators. Packets with paper-and-pencil teacher questionnaires were sent out or personally brought to the schools, and teachers were asked to fill in the questionnaires at home.

In the questionnaire, teachers were first asked to report about one of the classes they currently taught. To ensure random selection from the multiple classes these teachers could be expected to be currently teaching, they were prompted as follows: “Imagine it is Tuesday after the first class period. Which class will you be teaching next, according to your schedule? (note down the class label, e.g., 6a). In responding to the following items, please refer to this particular group of students.” In this section of the questionnaire, teachers were asked to report about a range of class characteristics including students’ age, class size, subject taught, weekly subject teaching hours in the class, and number of years they have been knowing the class (for descriptive statistics, see Table 1). Next, the teachers were asked to rate their emotions during teaching this class, as well as their judgments regarding this class’s performance, motivation, discipline level, and quality of the teacher–student relationships. Additionally, they were asked to report how important it was for them to achieve high levels of student performance, motivation, discipline, and high-quality teacher–student relationships (see below for the measures).

**TABLE 1:** Descriptive statistics for characteristics of all classes reported about.

	**Min**	**Max**	**Mean**	**SD**
Students’ age in class 1	10	22.50	14.09	2.47
Students’ age in class 2	10	21	13.96	2.47
Students’ age in class 3	6.50	20.50	14.40	2.41
Size of class 1	2	35	22.36	5.60
Size of class 2	2	32	22.93	5.31
Size of class 3	2	34	22.00	6.19
Weekly subject teaching hours in class 1	1	7	3.41	1.39
Weekly subject teaching hours in class 2	1	7	3.19	1.31
Weekly subject teaching hours in class 3	1	6	2.67	1.07
Number of years knowing class 1	0	8	1.52	1.35
Number of years knowing class 2	0	5	1.22	0.97
Number of years knowing class 3	0	6	1.60	1.28

In the next section of the questionnaire, teachers were asked to report about two more of their classes. To this end, they were prompted, respectively: “Imagine it is Wednesday/Thursday after the first class period. Which class will you be teaching next, according to your schedule? If this is the same class you have already reported about, please select the class that would come next in your schedule. Please note down the class label here (e.g., 6b/c).” Next, teachers were again asked to provide the same class characteristics about the classes as for the first class (such as class size and student age). In the last section of the questionnaire, teachers reported demographic information, including gender and age, as well as the state and school type they were currently teaching at.

Of the total of 244 teachers, 64/33/147 teachers reported about one, two, or three different classes, respectively. Missing data were treated by applying the full information maximum likelihood (FIML) approach.

### Measures

#### Teacher Emotions

We used the teacher emotions scales (TES; Frenzel et al., 2016) to assess teachers’ enjoyment, anger, and anxiety for the first class the reported about in the questionnaire. The TES contains four items each to assess these three discrete emotions. Sample items are, “In this class I enjoy teaching” for enjoyment, “In this class I often have reasons to get angry” for anger, and “When teaching this class, I am tense and nervous” for anxiety. Each scale demonstrated good internal consistency (Cronbach α’s for enjoyment/anger/anxiety = 0.92/0.87/0.83). For assessing enjoyment, anger, and anxiety in the second and third classes, single items were used (specifically, the sample items listed above) to not overwhelm teachers with the length of the questionnaire. Response options ranged from 1 (*strongly disagree*) to 5 (*strongly agree*).

#### Goal Attainment and Goal Importance Appraisals

To assess perceived goal attainment, we again used multi-item scales for the first class. Four items each were used to assess teacher judgments of student performance (e.g., “In this class there are many students who are quick on the uptake”), motivation (e.g., “In this class students are motivated”), and discipline (“In this class my teaching is often disrupted,” reverse coded). Response options again ranged from 1 (*strongly disagree*) to 5 (*strongly agree*). These scales have been applied successfully in prior research (Taxer and Frenzel, 2017) and also showed good internal consistencies in the present study (Cronbach α’s for performance/motivation/discipline judgments = 0.88/0.89/0.89). Goal attainment with respect to establishing high-quality teacher–student relationships in the class was assessed with a newly developed 5-item scale using a question format [e.g., “How would you describe your relationship with this class?” with response options ranging from 1 (*rather poor*) to 7 (*very good*)]. The scale showed high internal consistency (Cronbach α = 0.89). For goal attainment judgments in the second and third class, again, single items were used (specifically, the sample items listed above).

Goal importance appraisals were assessed only for the first class, through single items. Specifically, we asked teachers to rate “How important is it for you that as many students as possible in this class… comprehend the content and learn a lot (performance)/engage actively in class discussions (motivation)/abide with classroom goals and do not disrupt (discipline)/encounter you candidly and trustfully (teacher–student relationship quality)?” Items were answered on a scale from 1 (*comparably unimportant*) to 7 (*extremely important*).

### Data Analysis

Research Question 1a pertained to the amount of variance in teachers’ enjoyment, anger, and anxiety explained by teachers’ appraisals on a within-person level. In addressing it, we specified a multivariate two-level regression model using the software package Mplus (version 8; Muthén and Muthén, 1998-2017); employing the command “type = twolevel” and the MLR estimator, and the FIML method for treating missing data. This analysis used the single items pertaining to the three different classes the participating teachers had reported about. Thus, our two-level model involved classes on Level 1 and teachers on Level 2. We specified one single multivariate model with each of the three emotions as correlated outcomes, with the four goal attainment ratings as predictors on Level 1. We also included control variables, both on Level 1 (class size, student age, weekly subject teaching hours in the class, and number of years teachers have been knowing the class), and Level 2 (teacher gender, years of experience, and school type).

In addressing the links between teachers’ appraisals and emotions on an interindividual level (Research Question 1b), we specified a multivariate regression model using the R package lavaan (Rosseel, 2012; lavaan version 0.6–3), again employing the MLR estimator and the FIML method for treating missing data. This analysis used the manifest sums of multi-item scales for measuring emotions and goal attainment pertaining to the teachers’ first class addressed in the questionnaire.^3^ Again, we specified one single multivariate model, with each of the three emotions as correlated outcomes, and with the four goal attainment ratings as first-order predictors, alongside the control variables.

Finally, in addressing Research Question 2 pertaining to the additional contribution of goal importance appraisals, we added both goal importance appraisals and the goal attainment × goal importance interaction terms for each goal in this between-person multivariate regression model. All variables were *z*-standardized before analyses. This research question was addressed only in the context of the interindividual analyses because goal importance ratings were available only for the first class reported about by the teachers.

## Results

### Preliminary Analysis

Table 2 shows descriptive statistics for the study variables, including their bivariate correlations at the between-person and the within-person levels. For all variables that were assessed at the within-person level, we also inspected the intraclass correlations (ICCs), which represent the amount of variance between teachers relative to the total variance (i.e., between- plus within-person variance).

**TABLE 2:** Descriptive statistics for study variables.

	**Multi-item scales^1^ for Class 1**		**Single items for all three classes**				**Correlations**						
			
	**Mean**	**SD**	**Mean**	**SD_*between*_**	**SD_*within*_**	**ICC**	**JOY**	**ANG**	**ANX**	**ACH**	**MOT**	**DIS**	**REL**
Enjoyment (JOY)	4.00	0.88	4.08	0.70	0.80	0.07	1	–0.60	–0.65	0.42	0.60	0.47	0.74
Anger (ANG)	2.13	0.92	2.42	0.84	0.86	0.14	–0.73	1	0.49	–0.36	–0.46	–0.69	–0.49
Anxiety (ANX)	1.53	0.68	1.59	0.68	0.62	0.13	–0.70	0.67	1	–0.26	–0.45	–0.44	–0.65
Achievement attainment (ACH)	3.29	0.88	3.25	0.92	0.76	0.21	0.54	–0.49	–0.40	1	0.60	0.30	0.38
Motivation attainment (MOT)	3.46	0.77	3.48	0.70	0.73	0.05	0.71	–0.58	–0.56	0.73	1	0.40	0.59
Discipline attainment (DIS)	3.43	1.01	2.41	0.95	0.87	0.18	0.48	–0.71	–0.45	0.44	0.44	1	0.41
TSR attainment (REL)	5.76	1.01	5.69	0.80	0.86	0.15	0.79	–0.64	–0.61	0.49	0.70	0.41	1
Performance importance	6.04	0.94	—^1^	—^1^	—^1^	—^1^	0.28	–0.19	–0.19	0.17	0.27	0.25	0.29
Motivation importance	5.69	1.00	—^1^	—^1^	—^1^	—^1^	0.29	–0.14	–0.18	0.22	0.35	0.19	0.26
Discipline importance	5.71	1.15	—^1^	—^1^	—^1^	—^1^	0.09	0.07	0.03	0.04	0.03	–0.09	0.12
TSR importance	6.31	0.92	—^1^	—^1^	—^1^	—^1^	0.20	–0.05	–0.11	0.00	0.14	0.07	0.32

*Correlations on the within-person level are above the diagonal; correlations on the between-person level are below the diagonal. SD_*between*_ refers to the standard deviation across all teachers, SD_*within*_ refers to the pooled standard deviation within teachers. ^1^Importance appraisals were assessed with single items and only for Class 1. ICC, intraclass correlation; TSR, teacher–student relationship quality.*

Overall, teachers rather strongly endorsed the items assessing enjoyment during teaching. Anger and anxiety items were endorsed comparatively less. Teacher appraisals of the attainment of high student performance, motivation, and discipline were generally rather strongly endorsed, with mean levels well above 3 on the five-point answer scale. Likewise, teachers rather strongly endorsed goal attainment appraisals for teacher–student relationship quality, with a mean of close to 6 on the 7-point scale. These results were largely equivalent across the different measurement approaches taken in the present study, that is, as judged through the multi-item scale for the first class reported about, and through the average across the three enjoyment single items for three of their current classes. Regarding teacher ratings of the importance of each of the four goals, each of them was appraised as quite important.

The ICC can range between zero and 1, and the higher the ICC, the more a variable tends to be person-specific (in other words, there is little variance occurring within teachers across the different classes they refer to in their answers, but most variance occurs between teachers). The lower the ICC, the more the context plays a role (i.e., there is a lot of variance occurring within teachers across the different classes they refer to, and little variance occurs between teachers).

Intraclass correlations were quite low for teacher enjoyment (0.07) and student motivation goal attainment (0.05). As such, enjoyment and student motivation goal attainment appraisals were strongly context-specific. Slightly higher, implying yet still considerable within-teacher heterogeneity, were the ICCs for anger and anxiety, student discipline attainment, and teacher–student relationship quality attainment (ranging between 0.13 and 0.18). Highest ICCs were observed for teacher appraisals of student performance attainment (0.34). This suggests that there were systematic differences between teachers in judging their classes’ performance.

Replicating earlier findings from studies using the TES (e.g., Frenzel et al., 2016), correlations among emotions were medium-sized, with negative correlations between enjoyment and both anger and anxiety, and the latter being positively correlated. The correlations were small enough, though, to warrant conceptual separation of the three emotions. Correlations among the four goal attainment variables were consistently positive (ranging between 0.30 and 0.60 on the within-teacher level, and 0.41 and 0.73 on the between-teacher level), implying that if teachers felt successful in attaining one goal in one of their classes more so than in another class, they also tended to feel more successful at attaining the other goals in that class (within-teacher correlations). Also, this implies that some teachers seemed to generally feel more successful than others in attaining all of the four goals (between-teacher correlations). Correlations among the goal importance ratings were small to medium in size (ranging between 0.14 for the link between performance and discipline importance and 0.32 for the link between discipline and relatedness importance). This implies that teachers did not generally judge all of those goals as more or less important; instead, they seem to rank the importance of the goals quite differently. Overall, the correlations among the goal attainment and goal importance appraisals were small enough to preclude any severe multicollinearity (with the exception of the between-level link between goal attainment appraisals pertaining to motivation and achievement, which exceeded 0.70).

Furthermore, the attainment of each goal proved to be positively related with enjoyment and negatively related with anger and anxiety (ranging between |0.26| and |0.74| on the within-teacher level and between |0.40| and |0.79|on the between-teacher level), confirming our expectations that goal attainment appraisals would be positively linked with enjoyment, and appraisals of goal non-attainment would be positively linked with anger and anxiety. These bivariate correlations were again highly equivalent across the within- and between-person perspectives, and they were mostly medium in size, supporting the relevance of each of the goals for teachers’ emotional experiences. One single exception was the correlation between performance attainment and anxiety, which was comparably weak for anxiety on the within-teacher level (0.26). Goal importance appraisal/emotion correlations also were comparably weak, and so were goal importance/goal attainment correlations (Table 2).

### Within-Teacher Regression

Table 3 depicts the results of the multivariate multilevel regression analysis where all three emotions were simultaneously regressed on the four goal attainment appraisals, using single emotion and appraisal items pertaining to (up to) three different class ratings obtained per teacher. Attainment appraisals pertaining to student motivation, discipline, and teacher–student relationship quality showed significant individual positive links with teacher enjoyment, with teacher–student relationship quality attainment appraisals being clearly most relevant. Attainment appraisals pertaining to discipline and student motivation were significantly negatively linked with anger and anxiety, whereas discipline attainment was particularly relevant for anger, and teacher–student relationship quality attainment was particularly relevant for anxiety. Student performance attainment did not explain separate proportions of the within-teacher variance for any of the three emotions.

**TABLE 3:** Results from multilevel regression analyses (within-teacher analysis).

	**Enjoyment**		**Anger**		**Anxiety**	
**Variable**	**β**	**SE**	**β**	**SE**	**β**	**SE**
**Within-teacher level predictors**						
Performance attainment	0.09	0.04	–0.08	0.05	0.03	0.05
Motivation attainment	0.23**	0.06	–0.09	0.06	–0.09	0.06
Discipline attainment	0.20**	0.04	−0.61**	0.04	−0.26**	0.06
TSR attainment	0.60**	0.05	−0.23**	0.05	−0.55**	0.06
Students’ age	0.01	0.04	–0.03	0.04	0.08	0.05
Class size	0.00	0.04	0.03	0.04	–0.02	0.05
Number of years knowing the class	0.01	0.03	0.02	0.03	–0.01	0.04
Weekly subject teaching hours in class	0.06	0.03	0.07*	0.03	0.00	0.04
**Between-teacher level predictors**						
Teacher gender	0.21	0.15	0.02	0.12	0.15	0.11
Teaching experience	–0.13	0.14	–0.19	0.12	0.10	0.12
Dummy lowest track	0.03	0.19	0.16	0.13	–0.11	0.21
Dummy middle track	–0.07	0.19	0.28	0.15	–0.11	0.17
Dummy highest track	–0.10	0.21	0.02	0.18	–0.09	0.21
*R*^2^_*within*_*/R*^2^_*betweeen*_	0.47/0.08		0.45/0.14		0.39/0.07	

Significance level was set to **p < 0.01. TSR, teacher–student relationship quality.

None of the covariates were significantly linked with teachers’ emotional experiences, with one exception: the more hours per week a teacher taught a class, the more anger they would report to experience. Overall, when considering teacher attainment appraisals for all four proposed goals simultaneously, considerable amounts of within-teacher variance were explained (ranging between 0.39 for anxiety and 0.47 for enjoyment). As could be expected since the focus of this analysis lay on the explanation of the within-teacher variance, the proportions of explained variance on the between-teacher level were small (only teacher gender, years of experience, and school type were considered at the between-teacher level).

### Between-Teacher Regression

Table 4 depicts the results of multivariate regression analysis where all three emotions were simultaneously regressed on the four goal attainment appraisals, using the multi-item emotion and attainment appraisal scales, the single-item goal importance items, their interaction term (goal attainment × goal importance), and a range of control variables. Pertaining to Research Question 1b, teacher–student relationship quality attainment appraisals were significantly and quite strongly positively linked with enjoyment and negatively linked with anger and anxiety. In addition, there were significant links between attainment of motivation and enjoyment (positive) and with anxiety (negative). Attainment of discipline played an additional significant negative role for the emotions of anger and anxiety. Performance attainment appraisals did not have any predictive links for any of the emotions over and above the attainment appraisals pertaining to the other goals.

**TABLE 4:** Results from multivariate multiple regressions (between-teacher analysis).

	**Enjoyment**		**Anger**		**Anxiety**	
**Variable**	**β**	**SE**	**β**	**SE**	**β**	**SE**
Performance attainment	0.03	0.06	0.01	0.07	0.08	0.08
Motivation attainment	0.24**	0.08	–0.10	0.08	−0.27**	0.09
Discipline attainment	0.08	0.05	−0.49**	0.05	−0.19**	0.07
TSR attainment	0.58**	0.06	−0.44**	0.06	−0.43**	0.09
Performance importance	0.00	0.04	0.03	0.05	0.02	0.06
Motivation importance	0.05	0.05	0.05	0.05	0.01	0.06
Discipline importance	–0.03	0.05	0.06	0.05	0.08	0.06
TSR importance	–0.04	0.05	0.08	0.05	0.05	0.05
Performance attainment × importance	0.07	0.05	–0.08	0.05	–0.00	0.07
Motivation attainment × importance	–0.02	0.04	0.01	0.04	0.05	0.05
Discipline attainment × importance	0.12**	0.05	–0.06	0.05	–0.05	0.07
TSR attainment × importance	–0.01	0.03	–0.00	0.04	0.02	0.04
Students’ age	0.07	0.02	–0.03	0.02	–0.01	0.03
Class size	–0.00	0.01	0.02	0.01	–0.02	0.01
Number of years knowing the class	–0.09	0.03	0.06	0.03	0.02	0.04
Weekly subject teaching hours in class	–0.02	0.03	0.04	0.03	0.01	0.04
Teacher gender	0.05	0.09	0.01	0.09	0.04	0.11
Teaching experience	0.00	0.00	–0.01	0.00	–0.02	0.01
Dummy lowest track	–0.02	0.15	0.04	0.13	–0.05	0.23
Dummy middle track	0.01	0.15	0.04	0.13	–0.07	0.23
Dummy highest track	–0.06	0.14	0.02	0.12	–0.11	0.22
*R*^2^	0.72		0.68		0.46	

Significance level was set to **p < 0.01. TSR, teacher–student relationship quality.

Furthermore, in line with expectations regarding Research Question 2, there were no first-order effects of goal importance appraisals for any of the emotions. Furthermore, as expected, for enjoyment, the interaction for discipline was significant and positive, implying that teachers reported being particularly enjoying teaching if they deemed student discipline as important, coupled with judgments of high discipline levels in their classes. However, none of the other goal attainment × goal interaction showed any significant predictive power over and above the first-order effects of goal attainment.

Finally, none of the covariates were systematically linked with any of the emotions. Overall, considerable proportions of the between-teacher variability in emotions were explained by this model, ranging between *R*^2^ = 0.46 for anxiety, and *R*^2^ = 0.72 for enjoyment.

## Discussion

In the present study, we set out to test assumptions proposed by Frenzel and colleagues (Frenzel et al., 2009; Frenzel, 2014; Jacob et al., 2017), stating that teachers’ appraisals concerning the attainment and importance of teaching goals should be linked with their emotional experiences during teaching. While there had been scattered qualitative and quantitative evidence of the relevance of teaching goal attainment for teachers’ emotions, the present study was the first to systematically explore key propositions brought forward in Frenzel’s reciprocal model on teacher emotions (Frenzel et al., 2009; Frenzel, 2014; Jacob et al., 2017). Specifically, it provided new evidence to what degree teachers’ reported levels of enjoyment, anger, and anxiety levels were linked to their judgments of the attainment and importance of their students performing well, being motivated and engaged, demonstrating adequate discipline, and having a close relationship with their teachers. In so doing, we embraced a twofold assessment and analysis approach, exploring links between goal appraisals both on a between-teacher and on a within-teacher level.

### Findings on Within-Teacher Variability and Teaching Goal Importance

Despite not being at the core of our research questions, we considered our findings regarding within-teacher variability and goal importance ratings worthy of discussion. While there was substantial within-person variance of enjoyment, suggesting that enjoyment has a strong class-specific component, anger and anxiety ratings were more person-specific. For teacher anxiety, comparably high person specificity has been reported earlier (Frenzel et al., 2015). Additionally, we observed considerable within-teacher variability for all goal attainment appraisals, except for performance attainment. This finding implies that there were systematic differences between teachers in judging their classes’ performance. Those differences may be the result of person-specific biases (in the sense of generous vs. harsh general judgments of classes’ performance), but they may also be due to systematic differences between school types (with teachers from the lowest track judging of their classes as performing more poorly than teachers from the highest track). Such reasoning is supported by findings reported from large-scale scholastic competence studies, which have shown that, in Germany, classes vary systematically in their performance levels, and much of this between-class variability is due to school track (e.g., Maaz et al., 2008).

Furthermore, in and of themselves, our findings on teachers’ goal importance ratings seem noteworthy: across teachers, all four goals were considered highly important, while attaining high student motivation and discipline were considered less important than attaining performance and high-quality teacher–student relationships, and between-teacher variability was largest for motivation and discipline importance. Of course, the present study precludes assessing the importance of any other potential teacher goals, yet our findings support that the four goals proposed by Frenzel (2014), Jacob et al. (2017), and considered in this study indeed overall seem to be highly pertinent for many teachers.

### Findings on Goal Appraisal-Emotion Links

There were substantial bivariate links between each of the goal attainment appraisals and each of the three emotions under study, as judged both from a within- and from a between-teacher perspective. Based on the multivariate multiple regression analyses, on the within-teacher level, teachers reported enjoying teaching those classes more where they perceived their students as more motivated and disciplined as well as more closely attached to them. Anger and anxiety were both negatively linked with appraisals pertaining to the attainment of discipline and a high-quality relationship with students. On the between-teacher level, those teachers who reported more success in the attainment of motivation and high-quality teacher–student relationships reported higher enjoyment and lower anxiety and anger. Our second research question pertained to the additional variance which could be explained by goal importance appraisals. Counter to expectations, we found importance appraisals to be of minor relevance.

Our findings thus largely supported the relevance of the proposed goal attainment appraisals for teachers’ emotional experiences. It is worth noting, though, that both the between- and the within- teacher variance explained by the four goal attainment appraisals was lowest for the emotion of anxiety. Future research may consider exploring potential appraisal antecedents of teacher anxiety, beyond the attainment of the goals considered here, in more detail. Furthermore, the attainment of high student performance showed comparably weak bivariate links with each of the three emotions considered in this study. When the attainment of the three other goals was jointly taken into account, attainment of student performance was no longer significantly related to any of the three emotions. It is important to note that, particularly on the between-teacher level, goal attainment appraisals were rather highly correlated. Thus, conclusions regarding the relative importance of one appraisal over the other in predicting teacher emotions on the between-level have to be made with caution. In fact, the teacher judgments pertaining to the attainment of the four goals may also have lacked some validity in the sense of construct separability. It is not fully clear what teachers mentally refer to when they judge their classes as highly performing, motivated, or little disruptive and what they mean when agreeing that their students encountered them candidly and trustfully. One and the same student behavior may reveal the attainment (or non-attainment) of several of the goals. Indeed, we found that goal attainment appraisals were all positively intercorrelated, implying halo effects in judgments of classes, in the sense of some classes being generally judged as “better” with respect to the attainment of all goals relative to other classes. This may be one reason why the different goal attainment appraisals did not explain substantially separable sources of variance in the teachers’ emotions. However, these results may also imply that achieving high performance among students, in fact, is a function of motivation, discipline, and teacher–student relationship quality levels. In other words, high levels of student motivation, discipline, and teacher–student relationship quality may be a “means to the end” of high student performance. If this were the case, effects of performance would be mediated by effects of motivation, discipline, and relationship quality, which would explain why performance goal attainment appraisals were unrelated to teachers’ emotions, once the other goal attainment appraisals were considered. Exploring this in more detail, ideally with longitudinal designs that provide a more solid empirical basis for mediation hypotheses, seems to be a promising avenue for future research.

Furthermore, regarding the additional relevance of goal importance appraisals, we observed one effect that was in line with expectations, namely, a goal importance × goal attainment interaction for student discipline. This interaction implies that once teachers judged the discipline of their class as high, they enjoyed teaching the class more, and this effect was enhanced when they additionally judged student discipline as a particularly important teaching goal. However, it is important to note that, otherwise, we found no further evidence for the proposed relevance of goal importance appraisals. Of the 12 interaction terms tested, only one attained statistical significance. One explanation for these null findings could be that goal importance appraisals were measured with single items—thus with potentially lower reliability than the goal attainment appraisals, which were measured with multiple items—and hence, their explanatory power was deemed to be comparably low. In addition, teachers tended to endorse the importance of all four goals quite strongly, with means around 6 on the 7-point scale. Nevertheless, we could exclude that there were strong ceiling effects, as the range and variance of the goal importance appraisals were still considerable and comparable in size with the attainment appraisals.

### Theoretical and Practical Implications

An important strength of the present study is that it was explicitly designed to explore links between teachers’ goal appraisals and their emotions both on a between-teacher and on a within-teacher level (see Murayama et al., 2017; for a call to purposefully design studies to enable within-person analyses). Despite the apparent similarity of these two approaches, they, in fact, address quite different research questions. Through the within-teacher approach, we explored *when* a single teacher experiences most enjoyment, anger, and anxiety during teaching. This approach aligns well with the psychological theory we used to frame this research—appraisal theory—which postulates that emotions are aroused by individuals’ judgments pertaining to a situation. Findings from this approach allow developing intervention programs for individual teachers. In contrast, through the between-teacher approach, we explored *who* (across a population of teachers) experiences most enjoyment, anger, and anxiety during teaching. These findings do not allow for implications about intraindividual psychological functioning, but they are relevant from a policy perspective, for example, for teacher recruitment programs: They allow conclusions as to which individuals may be resilient against the psychological challenges involved in the teaching job, or potentially prone to burnout in the long run.

Importantly, even though relationships investigated in the between-person analysis and the within-person analysis are statistically independent, and it cannot be assumed that results from both approaches will necessarily converge (Hamaker et al., 2005; Voelkle et al., 2014; Fisher et al., 2018), the result patterns we obtained from the two approaches were highly equivalent in our study. This is in line with earlier findings on appraisal–emotion links, which also showed convergence across within- and between-person analysis approaches (e.g., Goetz et al., 2016; Murayama et al., 2017). By implication, future research on teacher emotions might rely on between-person designs only, which are typically less resource-consumptive and potentially provide more solid results: in between-person designs, participants can be asked to provide answers only with respect to a single context and not multiple, which in turn allows for more reliable measures (multi-item scales instead of single items).

An important limitation of the present study is that it was purely correlational. Any implications from such correlational data are valid only to the degree as the observed correlational patterns are interpreted in terms of underlying causal links, which is always problematic. For our data, we propose that teachers’ individual situational appraisals (for the within-person perspective) and their personal tendencies to judge their classes in one way or the other (for the between-person analysis) and teachers’ emotions are linked via reciprocal causation: On the one hand, emotions can be seen as drivers of perceptions and judgments, and on the other hand, appraisals are understood as determinants of emotions. While we do acknowledge both potential causal directions, we relied predominantly on the latter reasoning in terms of appraisal theory in our present study.

Another point worth mentioning is that the present study intendedly covered teachers from all three German major secondary school types (i.e., low, medium, and high track), the final sample turned out to not cover each school type to the same proportion; instead, a majority of teachers taught in high-track schools. The proposed appraisal–emotion links are generally thought to be basic human psychological phenomena and, as such, universal across contexts (see also Pekrun, 2006, for such universality assumptions regarding students’ appraisal–achievement emotion links). The present sample was still too small, though, to test for any potential moderating effects of school type. As such, it remains open to question, and future research, if the results presented herein might have been biased in any way due to oversampling of high-track teachers.

Different implications can be drawn from the between- and within-person findings of our study. Our most important finding from the between-person findings is that those teachers who manage to establish good relationships with their students seem to be better off emotionally during teaching. From this, we conclude that teacher recruitment should consider potential future teachers’ motivation to work with children and adolescents and their competencies in building relationships with children. High-quality relationships with students may also be conducive to achieving other classroom goals, including high student motivation and discipline (e.g., Pianta et al., 2012; Wubbels et al., 2014).

In turn, our key finding from the within-teacher analysis was that teachers’ emotions seem to be strongly linked with their subjective evaluations of student behaviors. By implication, teachers could be supported in modifying their emotional experiences through cognitive reappraisals. There is consistent evidence of the effectiveness of deliberate cognitive reappraisals for emotion regulation (e.g., Ochsner and Gross, 2008, for a review) and initial evidence of the trainability of emotion regulation through cognitive reappraisals (Denny and Ochsner, 2014). Again, teachers’ appraisals pertaining to the attainment of high-quality teacher–student relationships proved to be particularly relevant. It has been reported that teachers do spontaneously, but not very frequently, apply cognitive reappraisal as an emotion regulation strategy (Taxer and Gross, 2018). We know of no study that would have yet explored whether cognitive reappraisal trainings might be effective for teachers. Based on our findings, we deem this a promising road for future research and practice.

## Data Availability Statement

The datasets generated for this study are available on request to the corresponding author.

## Ethics Statement

Ethical review and approval was not required for the study on human participants in accordance with the local legislation and institutional requirements. The patients/participants provided their written informed consent to participate in this study.

## Author Contributions

AF, RP, and CR designed and implemented the research. AF and DF designed the computational framework and analyzed the data. AF took the lead in writing the manuscript. All authors provided critical feedback and helped shape the research, analysis, and manuscript. DF supported the formal manuscript completion.

## Conflict of Interest

The authors declare that the research was conducted in the absence of any commercial or financial relationships that could be construed as a potential conflict of interest.

## Footnotes

1 In earlier versions of this model, this goal was more broadly referred to as “social-emotional behavior,” while students’ discipline was suggested to play a key role in this context (Frenzel, 2014). In the present contribution, we chose to stick to the narrower conceptualization of this goal and explicitly speak of discipline behavior.

2 One teacher working at a lower-track school exceptionally also taught primary school kids and the third class he/she reported about was a grade 1 class.

3 Technically, we could have also used the aggregated single items for the three classes for this analysis. However, we had deliberately included multi-item scales for the first class in our study for the between-teacher analyses, as we deemed such multi-item scales to be more reliable and valid indicators of both the emotions and the goal attainment appraisals. In addition, goal importance appraisals were only available for the first class; thus, for answering Research Question 2, it was essential to run the analyses with particular reference to the first class.
